# Fahr’s Syndrome Secondary to Hypoparathyroidism Presenting With Neurological and Cardiac Manifestations

**DOI:** 10.7759/cureus.100506

**Published:** 2025-12-31

**Authors:** India Elliott, Shailesh Dalvi, King Leong, Stephanie Wong

**Affiliations:** 1 Cardiology, Wirral University Teaching Hospital NHS Foundation Trust, Liverpool, GBR; 2 Diabetes and Endocrinology, Wirral University Teaching Hospital NHS Foundation Trust, Liverpool, GBR

**Keywords:** basal ganglia calcification, case report, fahr’s syndrome, hypocalcaemia, hypoparathyroidism, non-sustained ventricular tachycardia, qt prolongation

## Abstract

Fahr’s syndrome is characterised by bilateral intracranial calcifications secondary to systemic or metabolic disease. We report a 56-year-old man with Fahr’s syndrome due to hypoparathyroidism who presented with collapse and seizure-like activity during alcohol consumption. Laboratory investigations demonstrated chronic hypocalcaemia with suppressed parathyroid hormone and hyperphosphataemia. Non-contrast CT revealed symmetrical calcifications of the basal ganglia and cerebellar dentate nuclei, confirming Fahr’s syndrome.

ECG on admission showed borderline QT prolongation (Bazett 479 ms; heart rate seventy-one beats per minute), consistent with repolarisation delay in hypocalcaemia. Lateral T-wave inversions were observed without chest pain, dynamic ST segment change, or troponin rise, and were interpreted as non-specific repolarisation abnormalities. Continuous telemetry captured several runs of non-sustained ventricular tachycardia (VT). Echocardiography demonstrated mildly reduced left ventricular systolic function with concomitant diastolic dysfunction.

The patient received intravenous calcium gluconate followed by oral calcium carbonate and active vitamin D (alfacalcidol), with ongoing rhythm surveillance using a cardiac monitor. On follow-up, there were intermittent supraventricular and ventricular ectopics and brief Wenckebach, but no sustained ventricular arrhythmias.

This case highlights the multi-system impact of chronic hypocalcaemia in hypoparathyroidism. While intracranial calcifications are irreversible, timely correction of calcium may ameliorate neurological symptoms and reduce arrhythmia risk. Clinicians should consider metabolic aetiologies in syncope with seizure-like activity and QT prolongation, and institute prompt biochemical correction alongside cardiac monitoring.

## Introduction

Basal ganglia calcification can arise from several causes. Primary familial brain calcification (Fahr’s disease) is idiopathic, usually autosomal dominant, and linked to mutations in SLC20A2, PDGFB, PDGFRB, and XPR1 [1]. By contrast, Fahr’s syndrome refers to secondary calcification due to an underlying condition; common causes include hypoparathyroidism, chronic kidney disease, infections, and toxins [2].

Although such calcifications may be incidental, their clinical significance increases when neurological features such as seizures, movement disorders, or psychiatric disturbances are present [3]. In hypoparathyroidism, chronic hypocalcaemia with hyperphosphataemia promotes calcium-phosphate deposition in the basal ganglia and dentate nuclei, with chronic kidney disease potentially exacerbating progression [4].

We present a patient with Fahr’s syndrome secondary to hypoparathyroidism, manifesting with both neurological and cardiac sequelae of calcium imbalance.

## Case presentation

A 56-year-old man presented after a witnessed loss of consciousness. Bystanders reported tonic-clonic movements. On recovery, he described jaw pain but no preceding aura, prodrome, or focal neurological deficit. His past medical history included epilepsy, chronic kidney disease, hypertension, hypoparathyroidism with hypocalcaemia, and long-standing alcohol and tobacco use.

The patient’s regular medications included colecalciferol (monthly), calcium carbonate 1 g three times daily, atorvastatin 20 mg nightly, bisoprolol 2.5 mg daily, felodipine 5 mg modified-release each morning, paracetamol 1 g four times daily, thiamine 100 mg three times daily, levetiracetam 500 mg twice daily, and alfacalcidol 1 μg three times daily.

On examination, he was haemodynamically stable (blood pressure 133/78 mmHg, heart rate 99 beats per minute, respiratory rate 18 per minute, temperature 36.4 °C, and capillary glucose 8.0 mmol/L). Neurological examination revealed normal cognition, motor strength, and sensation, with an acute non-sustained gaze-evoked nystagmus.

Laboratory investigations revealed persistent episodes of hypocalcaemia (corrected calcium 1.50-2.13 mmol/L; reference range 2.20-2.60 mmol/L), most pronounced during intervals between calcium and vitamin D supplementation, with suppressed parathyroid hormone (PTH) (0.3 pmol/L), hyperphosphataemia (1.72 mmol/L), and vitamin D insufficiency (43 nmol/L). Magnesium was borderline low (0.80 mmol/L). Creatinine ranged from 157-178 µmol/L. Cardiac biomarkers showed elevated NT-proBNP (991 ng/L) and a mild rise in troponin T (24 ng/L).

Serial biochemical results are summarised in Table 1, demonstrating fluctuating corrected calcium levels associated with changes in compliance and calcium supplementation.

**Table 1:** Serial biochemical trends and interventions. Serial corrected calcium levels demonstrate fluctuations associated with treatment and compliance. TDS = three times daily; μg = micrograms

Date	Corrected calcium (mmol/L)	Phosphate (mmol/L)	Vitamin D (nmol/L)	Parathormone (pmol/L)	Notes/intervention
Reference range	2.20-2.60	0.8-1.5	≥50 (sufficient)	1.6-6.9	Not applicable
Prior result	1.53	1.52	Not analysed	Not analysed	Poor compliance with alfacalcidol 3 μg and calcium carbonate 1 g TDS
Day 1 (admission)	1.76	Not analysed	Not analysed	Not analysed	Already on alfacalcidol 3 μg and calcium carbonate 1 g TDS
Day 2	2.64	1.72	43	0.3	Received IV calcium infusion
Day 3 (discharge)	2.27	1.31	Not analysed	Not analysed	Restarted alfacalcidol 3 μg daily and calcium carbonate 1 g TDS

**Figure 1:**
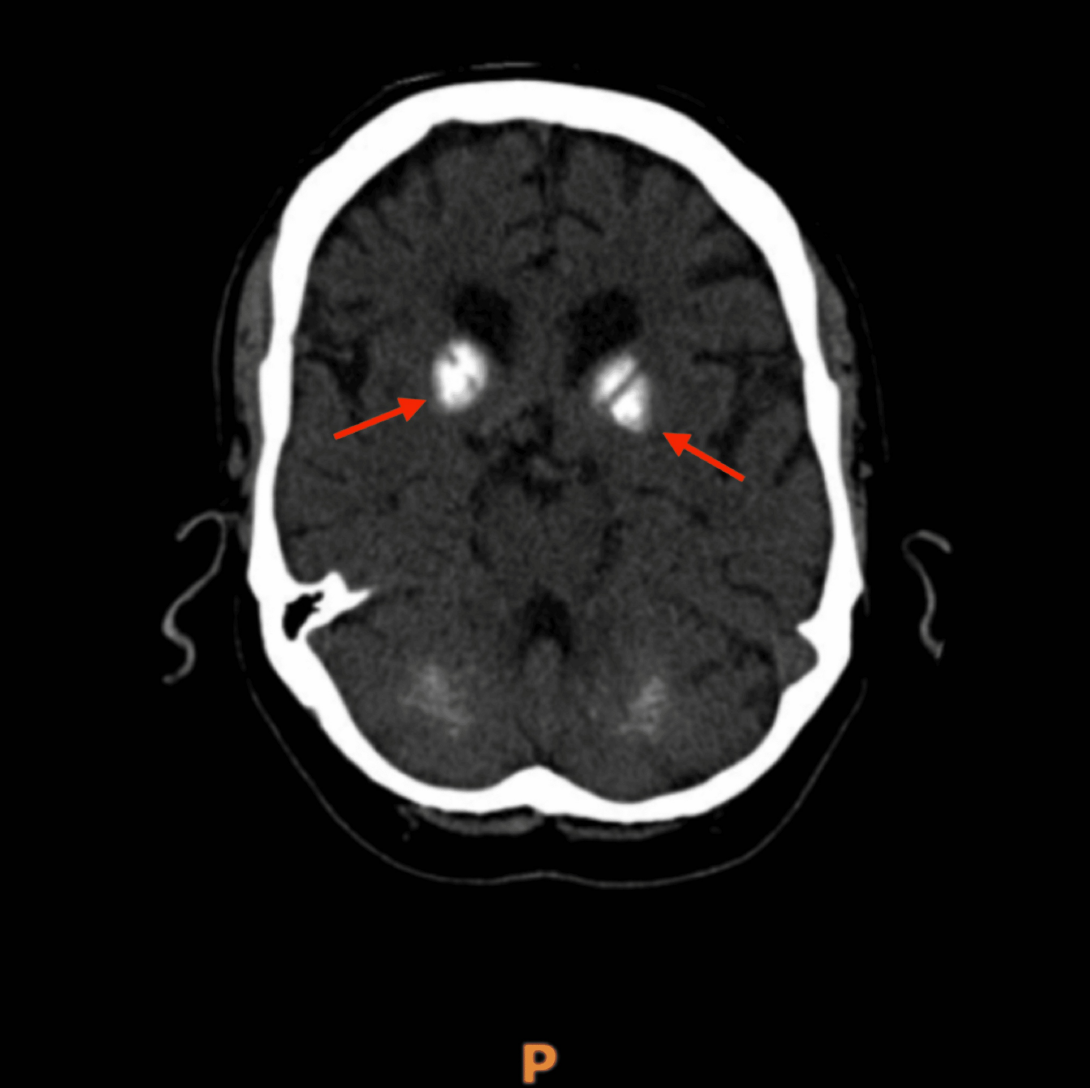
Axial non-contrast CT brain showing symmetric dense calcification in the bilateral basal ganglia (arrows), characteristic of Fahr’s syndrome.

**Figure 2:**
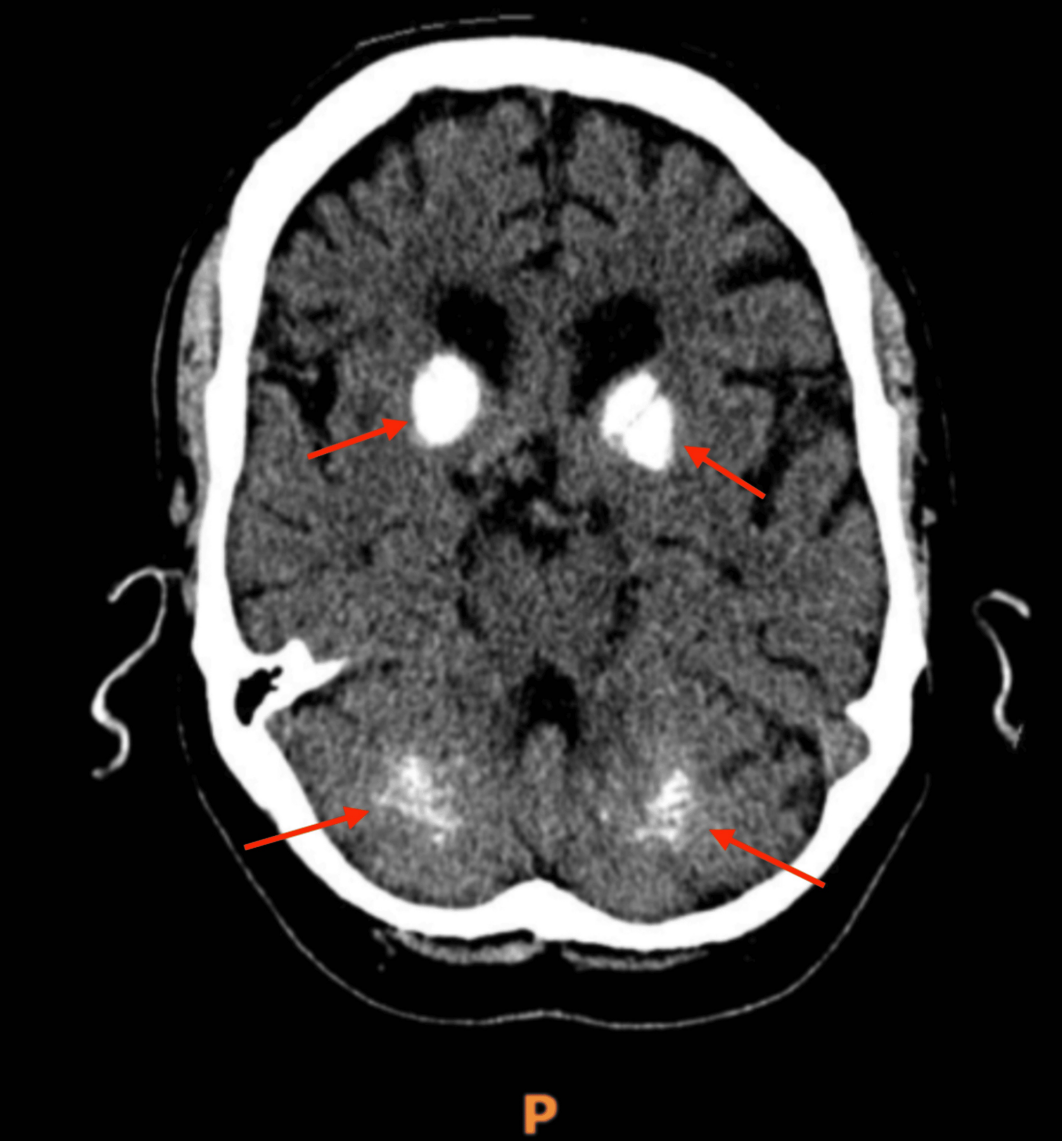
Axial non-contrast CT brain showing symmetric calcification of the bilateral basal ganglia and cerebellar dentate nuclei (arrows), consistent with Fahr’s syndrome.

Non-contrast CT of the brain (Figures 1-2) demonstrated symmetric calcification of the basal ganglia and cerebellar dentate nuclei, typical of Fahr’s syndrome.

**Figure 3:**
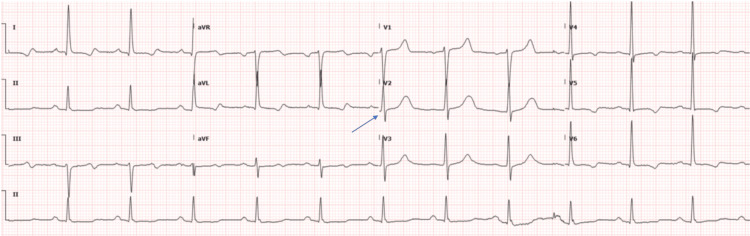
12-lead ECG demonstrating sinus rhythm with QT interval prolongation most apparent in precordial lead V2 (arrow). Paper speed 25 mm/second.

Electrocardiogram (ECG) (Figure 3) at presentation showed sinus rhythm, first-degree AV block with PR interval 202ms, borderline QT prolongation (Bazett 479 ms; heart rate seventy-one beats per minute). Lateral T-wave inversions were present without chest pain, dynamic ST-segment deviation, or dynamic troponin rise, and were considered non-specific repolarisation changes rather than an ischaemic pattern with mild ventricular hypertrophy.

**Figure 4:**
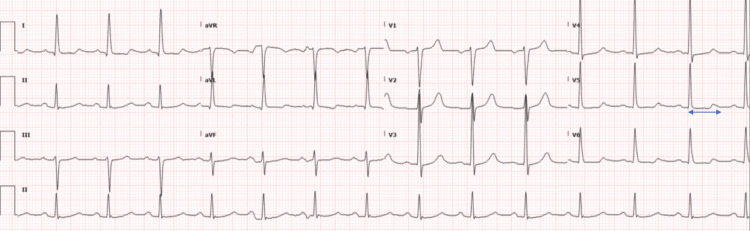
12-lead ECG demonstrating sinus rhythm with QT interval prolongation most apparent in precordial lead V5 (arrow). Paper speed 25 mm/second.

An ECG (Figure 4) from four months showed sinus rhythm, normal PR interval, near normal QTC 446ms (Bazett’s method) with mild ventricular hypertrophy.

Telemetry captured several runs of non-sustained ventricular tachycardia.

Transthoracic echocardiography demonstrated mildly reduced left ventricular systolic function (ejection fraction 49%).

Orthostatic blood pressure measurements and electroencephalography were not performed during this admission.

The patient received intravenous calcium gluconate followed by oral calcium and active vitamin D (alfacalcidol (One-Alpha)). Stabilising calcium levels was essential to reduce the risk of further seizures or arrhythmias.

He was monitored on telemetry and referred to cardiology, where an implantable loop recorder (ILR) was inserted for continuous, long-term heart rhythm monitoring. On follow-up, the device recorded occasional supraventricular and ventricular ectopy, brief episodes of Wenckebach phenomenon, and isolated couplets and triplets, with no sustained ventricular arrhythmia.

## Discussion

Fahr’s syndrome represents a spectrum of multiorgan involvement arising from abnormal calcium regulation and deposition, most prominently within the basal ganglia and cerebellum. Neurological manifestations of intracranial calcification include seizures, syncope-like episodes, and extrapyramidal features such as Parkinsonism or chorea, with recognised neuropsychiatric symptoms ranging from mood disturbance to psychosis [5-7]. The presence of primary familial basal ganglia calcification is referred to as Fahr’s disease, whereas Fahr’s syndrome describes secondary calcification resulting from disordered calcium-phosphate metabolism [8].

Our differential diagnosis was either seizure in the setting of brain calcified lesions or convulsive syncope related to ventricular arrhythmia, in light of non-sustained ventricular tachycardia on telemetry. This finding emphasises the overlap of neurological and cardiac manifestations in hypocalcaemia.

Distinguishing epileptic seizures from convulsive syncope can be challenging in hypocalcaemia, where both neuronal hyperexcitability and cardiac arrhythmia may coexist. Seizures are typically associated with a post-ictal phase, tongue biting, incontinence, or focal deficits, whereas syncope is characterised by abrupt loss of consciousness with rapid recovery. In this case, the absence of post-ictal features and the presence of non-sustained ventricular tachycardia favoured a cardiac mechanism, with symptom resolution following calcium replacement supporting convulsive syncope [5,9].

Hypocalcaemia is a recognised pro-arrhythmic state due to altered myocardial excitability [9,10]. Although marked QTc prolongation is a well-described feature of hypocalcaemia, our patient demonstrated a borderline QTc prolongation rather than overt QT prolongation. Nevertheless, ventricular arrhythmias, including non-sustained ventricular tachycardia, may occur in the absence of significant QT prolongation, particularly in the context of acute electrolyte disturbance [9]. Mild hypomagnesaemia may have contributed to the arrhythmic risk, as magnesium deficiency disrupts calcium handling and myocardial repolarisation, and can exacerbate the electrophysiological effects of hypocalcaemia [9,10]. The patient’s chronic hypocalcaemia was likely exacerbated acutely by ongoing alcohol use, contributing to electrolyte instability and precipitating arrhythmia and seizure-like activity.

In this case, the temporal association between hypocalcaemia and episodes of non-sustained ventricular tachycardia, together with resolution following correction of calcium levels, supports a causative relationship. Similar cases describing hypocalcaemia-associated ventricular arrhythmias presenting with seizure-like activity have reported complete resolution following calcium replacement [9]. Calcium replacement remains central to management, as biochemical correction improves neuronal stability and may reduce seizure burden, though anti-epileptic therapy is often still required [5].

Chronic hypocalcaemia also impairs cardiac contractility, producing both systolic and diastolic dysfunction that can manifest as heart failure or cardiomyopathy. Several reports describe improvement after calcium and active vitamin D replacement [10]. In this case, transthoracic echocardiography demonstrated mildly reduced left ventricular systolic function (ejection fraction 49%), supporting cardiac involvement in the context of chronic hypocalcaemia. However, additional contributory factors to the reduced contractility included the patient’s long-term alcohol use, smoking history, hypertension, and chronic kidney disease.

Non-contrast CT is the imaging gold standard for detecting intracranial calcification and is the most sensitive modality in suspected Fahr’s syndrome. MRI is less sensitive to calcification but can delineate coexisting structural abnormalities, such as white matter disease or cortical atrophy [11]. In our patient, the intracranial calcifications were considered chronic and unlikely to account for the acute presentation, instead reflecting long-standing disordered calcium-phosphate metabolism predisposing to neurological manifestations.

The principal differential diagnosis is primary familial brain calcification (Fahr’s disease) - a genetic disorder typically presenting earlier in life, without biochemical abnormalities or hypocalcaemia [1,8]. Our patient had no family history of primary familial brain calcification, and genetic testing was not performed. Additionally, our patient exhibited suppressed PTH and hyperphosphataemia, consistent with secondary calcification due to hypoparathyroidism.

Chronic kidney disease may also contribute through disordered calcium-phosphate metabolism, though basal ganglia involvement in that context is usually accompanied by vascular calcification [2]. Pseudohypoparathyroidism was excluded by the low PTH level [12]. Infectious or toxic causes were considered unlikely given the symmetrical basal ganglia distribution and chronic biochemical abnormalities [13].

In this case, the combination of long-standing hypocalcaemia, suppressed PTH, and symmetrical basal ganglia calcification supported a diagnosis of Fahr’s syndrome secondary to hypoparathyroidism rather than alternative causes. The patient's convulsive episodes, cardiac arrhythmias, and neuroimaging findings are best understood as systemic manifestations of chronic hypocalcaemia, reflecting its effects on neuronal excitability, myocardial electrophysiology, and intracranial calcium deposition.

Management focuses on correction of the underlying biochemical disturbance with oral calcium and active vitamin D analogues such as alfacalcidol or calcitriol [5]. Given the arrhythmic risk associated with QT prolongation, continuous telemetry and ongoing rhythm surveillance are recommended in patients presenting with syncope or unexplained collapses [9]. Normalising calcium levels reduces both seizure frequency and arrhythmia risk, although patients with established epilepsy may still require antiepileptic medication [5].

## Conclusions

Although the CT brain demonstrated findings consistent with Fahr’s syndrome, it remains uncertain whether this was the underlying cause of the seizures or a manifestation of persistent hypocalcaemia. The presence of a borderline prolonged QT interval was less likely to have caused the convulsive syncope in this case, as no further QT worsening or sustained ventricular arrhythmias were observed on cardiac monitoring. In this case, the patient had a background of chronic hypocalcaemia and a documented history of epilepsy, with acute metabolic deterioration likely precipitated by chronic alcohol use. This acute worsening of hypocalcaemia provides the most plausible trigger for the observed cardiac arrhythmia and convulsive syncope activity. The cerebral calcifications identified on CT imaging are likely chronic and incidental, reflecting long-standing hypocalcaemia rather than the direct cause of the acute presentation. There was no documented drop in blood pressure, although a transient haemodynamic contribution cannot be entirely excluded.

Fahr’s syndrome secondary to hypoparathyroidism can produce both neurological and cardiac complications. In this case, hypocalcaemia likely precipitated seizure-like activity and non-sustained ventricular tachycardia, emphasising the importance of considering metabolic causes in patients presenting with collapse and repolarisation abnormalities. Hypocalcaemia can lead to both neurological and cardiac manifestations, including seizure-like episodes and arrhythmias. Fahr’s syndrome should be suspected when basal ganglia calcifications coexist with calcium-phosphate abnormalities. While intracranial calcifications are irreversible, the cardiac manifestations of hypocalcaemia are often reversible with prompt biochemical correction.
